# Draft Genome Sequences of *Campylobacter fetus* subsp. *venerealis* bv. venerealis Strain B6 and bv. intermedius Strain 642-21

**DOI:** 10.1128/genomeA.00943-14

**Published:** 2014-10-02

**Authors:** Roberto A. Barrero, Paula Moolhuijzen, Léa Indjein, Bronwyn Venus, Gabriel Keeble-Gagnère, John Power, Matthew I. Bellgard, Ala E. Lew-Tabor

**Affiliations:** a Centre for Comparative Genomics, Murdoch University, Perth, Australia; b School of Veterinary Science, the University of Queensland, Gatton, Queensland, Australia; c Queensland Alliance for Agriculture and Food Innovation, the University of Queensland, St. Lucia, Queensland, Australia; d Zoetis, Parkville, Australia

## Abstract

*Campylobacter fetus* subsp. *venerealis* is an important venereal pathogen. We sequenced the genomes of *Campylobacter fetus* subsp. *venerealis* bv. venerealis strain B6 and bv. intermedius strain 642-21. The genetic variability of these Australian strains will facilitate the study of mechanisms of geographical adaptation of these pathogens that impact livestock.

## GENOME ANNOUNCEMENT

*Campylobacter fetus* is an important veterinary pathogen worldwide. This species comprises two closely related subspecies, *C. fetus* subsp. *fetus* and *C. fetus* subsp. *venerealis* (1). The former is a generalist subspecies that colonizes the intestinal and genital tracts of multiple host including sheep, cattle, birds, and humans (2). The latter subspecies, *C. fetus* subsp. *venerealis*, is normally restricted to colonization of the genital tract in ruminants (3). *C. fetus* subsp. *venerealis* is the causative agent of bovine genital campylobacteriosis; asymptomatic in bulls, the disease is spread to female cattle and causes infertility and epidemic abortions (4). In a recent study, *C. fetus* subsp. *venerealis* was shown to contain unique lipopolysaccharide production and type IV secretion machineries as compared to *C. fetus* subsp. *fetus* (5). Targeted mutational inactivation demonstrated the role of lipopolysaccharide biosynthesis genes in the modulation of virulence and host range (5). The evolutionary interplay between microbial pathogens and their hosts is a continual process of adaptation, manifested by genomic variation of host adaptation factors, and by the gain and loss of genes via horizontal gene transfer. Thus, further understanding of local and regional adaptations of these pathogens is necessary to capture the genetic diversity of pathogens isolated from distinct geographic locations worldwide.

Here we report the sequencing of two *C. fetus* subsp. *venerealis* biovar genomes isolated in Australia, *C. fetus* subsp. *venerealis* bv. venerealis strain B6 and *C. fetus* subsp. *venerealis* bv. intermedius strain 642-21. Sequencing was performed on an Illumina/GAII platform and generated 23,021,027 and 22,819,299 paired-end reads with an insert size of 320 bp for *C. fetus* subsp. *venerealis* bv. venerealis strain B6 and *C. fetus* subsp. *venerealis* bv. intermedius strain 642-21, respectively. The resulting libraries were quality trimmed using an in-house script and then assembled using ABySS (6, 7). The assembly yielded 80 and 126 scaffolds encoding 1,945,080 bp and 1,942.51 bp for the *C. fetus* subsp. *venerealis* bv. venerealis strain B6 and *C. fetus* subsp. *venerealis* bv. intermedius strain 642-21, respectively. Preliminary comparative analyses show that *C. fetus* subsp. *venerealis* bv. venerealis strain B6 encodes a highly conserved counterpart of the known ATCC 19438 pathogenicity island (PAI) encoding type IV secretory genes (8). In contrast, *C. fetus* subsp. *venerealis* bv. intermedius strain 642-21 appears to either lack or contain a highly divergent PAI. However, more extensive analyses are needed to confirm that *C. fetus* subsp. *venerealis* bv. intermedius strain 642-21 may have a distinct or divergent type IV secretory machinery and/or that this may represent an environmental adaptation.

### Nucleotide sequence accession numbers.

The whole-genome shotgun assemblies for the *C. fetus* subsp. *venerealis* bv. venerealis strain B6 and *C. fetus* subsp. *venerealis* bv. intermedius strain 642-21 were deposited at GenBank/DDBJ/EMBL under the accession numbers AJMC00000000 and AJSG00000000, respectively.
